# X-ray diffraction images for two membrane protein crystals presenting high anisotropy; the *B. subtilis* ABC transporter BmrA and the *S. pneumoniae* NADPH oxidase

**DOI:** 10.1107/S2414314625005917

**Published:** 2025-07-04

**Authors:** Veronica Zampieri, Annelise Vermot, Michel Thepaut, Isabelle Petit-Hartlein, Franck Fieschi, Pierre Falson, Vincent Chaptal

**Affiliations:** a EMBL Grenoble, 71 Avenue des Martyrs, Grenoble, France; b Univ. Grenoble Alpes, CNRS, CEA, Institut de Biologie Structurale Grenoble France; c Institut Universitaire de France Paris France; d Molecular Microbiology & Structural Biochemistry, UMR5086, CNRS University Lyon-1 France; University of Manchester, United Kingdom

**Keywords:** membrane protein, diffraction anisotropy

## Abstract

Diffraction datasets of membrane protein crystals were used to assess the diffraction anisotropy phenomenon.

## Introduction

Diffraction anisotropy is a phenomenon more prevalently encountered with membrane protein crystals (Robert *et al.*, 2017 ▸), which results in artefacts in electron density maps making them difficult to interpret and prone to error in model building. Our previous study suggests that anisotropy would be linked to collinearity of secondary structure elements that cause stronger diffraction peaks in the 5 Å resolution limit area (Martin *et al.*, 2021 ▸). This collinearity of secondary structure elements is always observed in membrane proteins where *trans*-membrane segments are normal to the membrane plane. The data presented here (Table 1 ▸) are for two membrane proteins showing typical low resolution and strong anisotropy that represented a major challenge in their structure solving. It is believed that the diffraction anisotropy present in the data is in fact the result of two unrelated phenomena. There is (i) a difference in resolution limits linked to the crystal packing that complicates the definition of a single resolution limit for the dataset, and (ii) a difference in intensity falloff in 3D compared to the empirical curve first described by Popov & Bourenkov (2003 ▸), leading to the idea that membrane proteins don’t follow the empirical law derived from highly diffracting crystals of soluble proteins. The data presented here can be used by methods developers to test various hypotheses on how to handle such datasets.

### The *B. subtilis* multidrug ABC transporter BmrA

**Figure 1:**
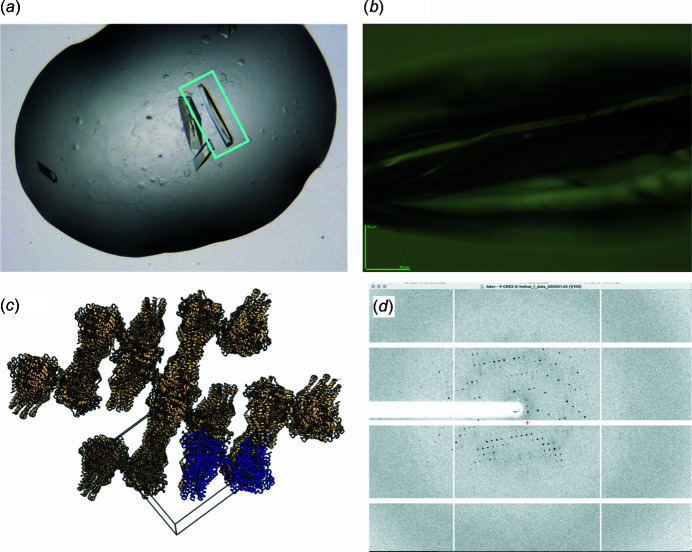
ABC transporter BmrA. (*a*) A crystal in a hanging drop. The diffracting crystal is in the cyan rectangle. (*b*) The same crystal viewed from the PX2 beamline camera, probably before helicoidal data collection. (*c*) The crystal packing and unit cell. The asymmetric unit is shown in blue. (*d*) Typical diffraction image.

The inactive catalytic mutant E504A was used to trap the transporter in the outward-facing conformation in complex with ATP-Mg^2+^. The transporter is a homodimer and crystallized in the *P*2_1_ space group with two homodimers in the asymmetric unit. The solvent content is around 69%, typical for a membrane protein crystallized in detergent. The crystal that gave the highest resolution data was a large, long rod (Fig. 1 ▸). The crystal was illuminated using a microfocus beam on beamline PX2 (Duran *et al.*, 2013 ▸) at SOLEIL using two collection strategies. First it was collected at a single spot at 5% transmission for a low-dose data collection. This data set is included in the Zenodo deposition named ‘V-CK63-8-ld_1_data_xxxxxx.h5’. Second, diffraction data were collected with a helical crystal translation strategy at nearly 100% transmission after moving the crystal so that the beam was hitting a different (previously unexposed) part of the crystal compared to the previous datasets; this data set is included in the Zenodo deposition named ‘V-CK63-8-helical_1_data_xxxxxx.h5’. For information, by using a microfocus beam on such a large crystal we hoped to identify subdomains of better diffracting quality. Unfortunately, we were not able to identify any as judged by visual inspection of the diffraction patterns.

The structure of BmrA solved and refined using only the helical crystal translation data from this crystal has been published as part of the article by Chaptal *et al.* (2022 ▸), with a detailed material and methods section on structure solving and model building, which were challenging for this project.

### The *S. pneumoniae* NADPH oxidase

**Figure 2:**
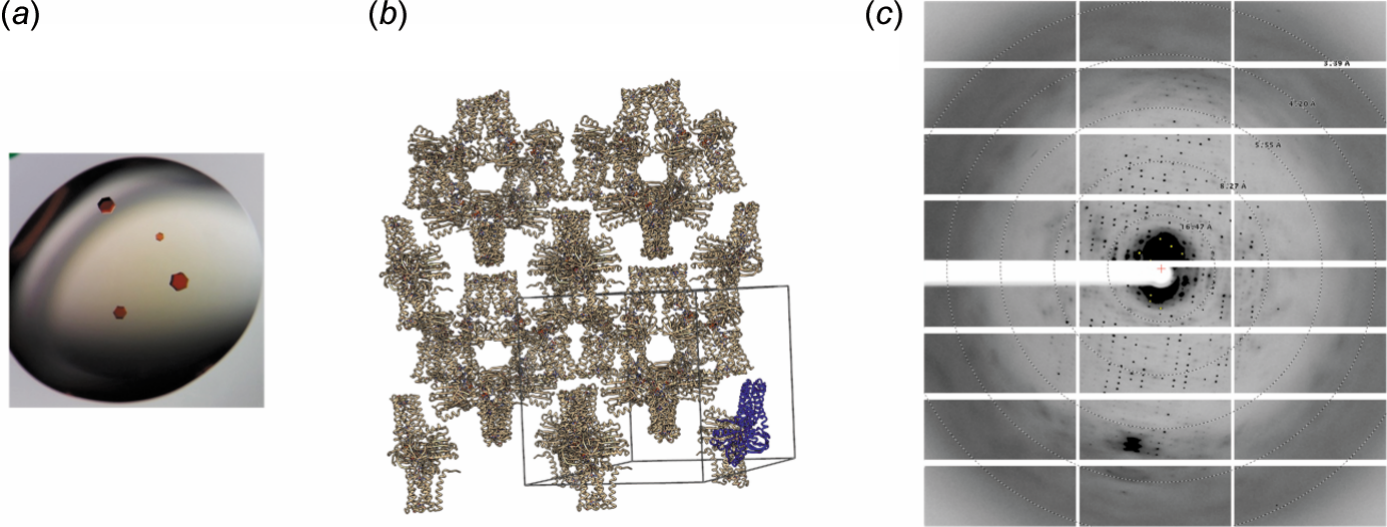
NADPH oxidase SpNOX. (*a*) A typical spNOX crystal in hanging drops. (*b*) Crystal packing and unit cell for SpNOX. The asymmetric unit is shown in blue. (*c*) Typical diffraction image.

A F397W mutant of the *S. pneumoniae* NADPH oxidase (SpNOX) was crystallized with the *trans* membrane domain complexing two hemes and the de­hydrogenase (DH) domain complexed with FAD. The full-length F397W SpNOX in detergent was crystallized as a monomer in space group *P*6_4_22 with a high solvent content of 76%. The typical crystal shape was a nice hexagonal firebrick (Fig. 2 ▸). More than 500 crystals were exposed to X-rays on the MASSIF-1 automatic beamline (Bowler *et al.*, 2015 ▸) at ESRF, Grenoble and 187 diffraction datasets were recorded and processed; among them only 32 reached resolutions better than 4 Å. A high-resolution structure is also available for the DH domain alone (https://doi.org/10.2210/pdb8qq5/pdb) (Petit-Hartlein *et al.*, 2024 ▸) that was used to solve our structure using molecular replacement. Two search models were used, first the DH domain was placed, then a model of the transmembrane domain made on the basis of multiple sequence alignment of NOX homologs and validated by structural alignment on CsNOX, another procaryotic NOX (Magnani *et al.*, 2017 ▸). This high-resolution structure of the DH domain was also used during our model refinement as a reference template during model building. For the final refinement we used software dedicated to low resolution structures modelling, *ISOLDE *(Croll, 2018 ▸) and *LORESTR *(Kovalevskiy *et al.*, 2016 ▸) as described in the original paper (Petit-Hartlein *et al.*, 2024 ▸).

The structure of SpNOX solved and refined using data from this crystal was published as part of this article (Petit-Hartlein *et al.*, 2024 ▸), with a detailed material and methods section on structure solving and model building, which as mentioned above was challenging for this project.

## Supplementary Material

DOI for imageCIF 8QQ7.cif: https://doi.org/10.5281/zenodo.14901515

Metadata imgCIF file. DOI: 10.1107/S2414314625005917/he4560img1.cif

Metadata imgCIF file. DOI: 10.1107/S2414314625005917/he4560img2.cif

Metadata imgCIF file. DOI: 10.1107/S2414314625005917/he4560img3.cif

## Figures and Tables

**Table 1:** Experimental details

Raw data	V-CK63-8-helical	V-CK63-8-ld	SpNox-Sp49-B3-B
DOI	https://doi.org/10.5281/zenodo.14894181	https://doi.org/10.5281/zenodo.14894181	https://doi.org/10.5281/zenodo.14901515
Data archive	Zenodo	Zenodo	Zenodo
Data format	HDF5	HDF5	CBF
			
Beamline	SOLEIL PX2	SOLEIL PX2	ESRF ID30
Detector	Eiger S 9M	Eiger S 9M	Pilatus3 2M
Radiation type	X-ray	X-ray	X-ray
Wavelength (Å)	0.9801	0.9801	0.9660
Beam centre (mm)	-111.2, 114.5	-111.2, 114.5	-130.2, 146.8
Detector axis	-Z	-Z	-Z
Detector distance (mm)	300.05	300.05	500.28
Pixel size (mm)	0.075 × 0.075	0.075 × 0.075	0.172 × 0.172
No. of pixels	3110 × 3269	3110 × 3269	1475 × 1679
No. of scans	1	1	1
Exposure time per frame (s)	0.025	0.025	1.7
Scan axis	ω, X	ω, X	ω, X
Start angle, increment per frame (°)	0.00, 0.10	0.00, 0.10	0.00, 0.20
Scan range (°)	360.00	360.00	260.00
No. of frames	3600	3600	1300
PDB code	6r72	–	8qq7
